# Evaluation of Boric Acid Treatment on microRNA‐127‐5p and Metastasis Genes Orchestration of Breast Cancer Stem Cells

**DOI:** 10.1007/s12011-024-04274-6

**Published:** 2024-07-04

**Authors:** Tuğba Semerci Sevimli, Aynaz Ghorbani, Fidan Gakhiyeva, Aliakbar Ebrahimi, Hamed Ghorbanpoor, Burcugül Altuğ, Fulya Buge Ergen, Zarifa Ahmadova, Merve Nur Soykan, Emre Tufekcioglu

**Affiliations:** 1 Cellular Therapy and Stem Cell Production, Application, and Research Center (ESTEM), Eskisehir Osmangazi University, Eskisehir, 26040 Turkey; 2 Department of Biomedical Engineering, Faculty of Engineering and Architecture, Eskisehir Osmangazi University, Eskisehir, 26040 Turkey; 3 Department of Surgery, Medical Faculty Mannheim, Heidelberg University, 68167 Mannheim, Germany; 4 Department of Industrial Design, Faculty of Architecture and Design, Eskisehir Technical University, Eskisehir, 26555 Turkey

**Keywords:** Breast cancer, Cancer stem cell, Boric acid, Metastasis, miR-127-5p

## Abstract

Coregulation of microRNAs (miRNAs) and cancer stem cells (CSCs) is very important in carcinogenesis. miR-127-5p is known to be downregulated in breast cancer. In this study, we aimed to investigate how boric acid (BA), known for its previously unstudied anti-cancer properties, would affect the expression of miR127-5p and genes responsible for breast cancer stem cells (BC-SCs) metastasis. BC-SCs were isolated from human breast cancer cells (MCF-7) by immunomagnetic cell separation and characterized with flow cytometry and sphere formation. The viability of BC-SCs and the determination of its IC50 value in response to boric acid (BA) were assessed via the MTT assay. Boric acid exhibited dose- and time-dependent inhibition of cell viability in cells. The IC50 doses of boric acid in MCF-7 cells and BC-SCs were 45.69 mM and 41.27 mM, respectively. The impact of BA on the expression of metastatic genes and miR127-5p was elucidated through RT-qPCR analysis. While the expression of the COL1A1 (*p* < 0.05) and VIM (*p* < 0.01) was downregulated, the expression of the miR-127-5p, ZEB1 (*p* < 0.01), CDH1 (*p* < 0.05), ITGB1 (*p* < 0.05), ITGA5 (*p* < 0.05), LAMA5 (*p* < 0.01), and SNAIL (*p* < 0.05), was up-regulated in dose-treated BC-SCs (*p* < 0.001) to the RT-qPCR results. Our findings suggest that boric acid could induce miR-127-5p expression. However, it cannot be said that it improves the metastasis properties of breast cancer stem cells.

**Graphical Abstract:**

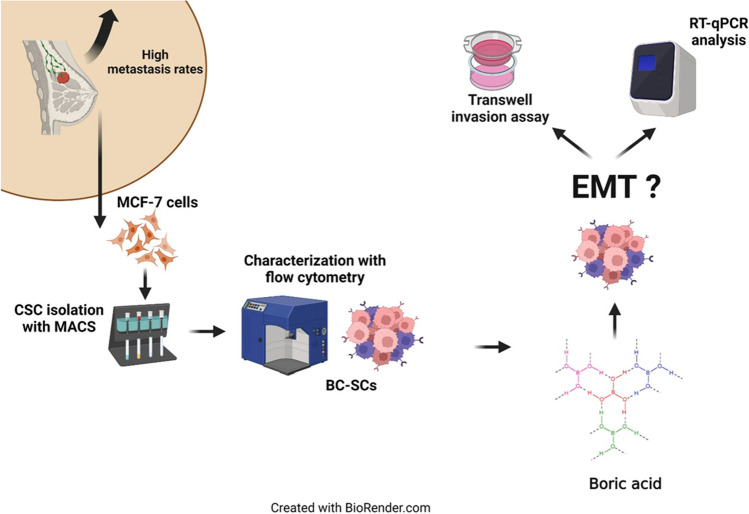

## Introduction

MicroRNAs (miRNAs) are small RNA molecules, typically 19 to 25 nucleotides long, which play a crucial role in regulating gene expression by inducing post-transcriptional silencing of target genes. A single miRNA can modulate the expression of numerous mRNA targets, thereby influencing various genes involved in interconnected functional pathways [1]. Numerous miRNAs have been discovered to possess either tumor-suppressive or oncogenic properties. The revelation of the extensive genomic complexity of cancer cells has underscored the importance of these small, non-coding RNAs. Through the characterization and cloning efforts, over 1000 miRNAs have been identified, highlighting their significant regulatory roles in cancer biology [2, 3].

miR-127 was one of the earliest identified miRNAs to be epigenetically regulated, with its expression often silenced in human cancer cell lines and primary tumors. Extensive research has revealed its tumor-suppressive role in various cancers such as stomach, pancreatic, ovarian, and esophageal cancers, as well as hepatocellular carcinoma and osteosarcoma. However, conflicting findings exist, with some studies, particularly those focusing on glioblastoma and lung cancer, suggesting an oncogenic function of miR-127 [4].

In breast cancer, miRNA-127 has been demonstrated to act as a tumor suppressor. Low expression levels of miRNA-127 are associated with advanced clinical stages, reduced overall survival, and the presence of lymph node metastases, all of which serve as independent prognostic factors for breast cancer [5, 6].

Boron is a non-metallic element in the form of Borax (sodium tetraborate; Bx) and boric acid (BA), with boric acid being the predominant form of boron in plasma. It is plentiful in various foods, mainly fruits and seeds [7]. Boron in the diet also passes into the colon, which is found as indigestible boron [8]. Studies examining boron’s effect on breast cancer have shown its antiproliferative and antiapoptotic effects [9–11]. Our previous study also showed that it affects some DNA Double-strand break (DSB) repair genes in breast cancer stem cells [12].

In this study, we aimed to examine the effect of BA on metastasis genes and miRNA127-5p expression. There is no study on metastasis genes and miRNA127-5p expression in breast cancer stem cells. We believe that with the results obtained, we will contribute to research on the relationship between breast cancer metastasis and miR127-5p.

## Materials and Methods

### MCF-7 Cell Line Culture

Human breast cancer cells (MCF-7, ATCC® HTB-22™, USA) were used in the study. MCF-7 cells were cultured in RPMI-1640 medium (Invivogen, USA) supplemented with 10% fetal bovine serum (Invivogen, USA) and 1% penicillin/streptomycin (Invivogen, USA) in a humidified atmosphere of 95% air–5% CO_2_ at 37 °C. It was passed into subcultures when the cells reached 70% cell density (approximately 2–3 days).

### Breast cancer stem cell (BC-SC) Isolation and Characterization

BC-SCs were isolated from MCF-7 cells by immunoselection with positive CD44 (Biolegend, USA); CD117 (Biolegend, USA); CD133 (Biolegend, USA); CD326 (Biolegend, USA); and ABCG2 (Biolegend, USA) expressions in our previous study [12]. The confirmation of the BC-SCs characters was shown with flow cytometry analysis. Cell suspensions were read on an AGILENT NovoCyte D3005 (Agilent, USA, Santa Clara) device and analyzed using the software program.

### Mammosphere Assay

BC-SCs were resuspended at 1 × 10^6^ cells/mL density and placed in culture with 3D Tumorsphere Medium XF (PromoCell, Germany). The cells were incubated for 4–10 days, with daily observation under a microscope to track tumorsphere formation. Upon tumorsphere formation, the medium was replenished every 3–4 days, and passaging was conducted until the tumorspheres displayed a discernible dark center, indicative of maturation.

### Cell Cytotoxicity Assay

#### Boric Acid

A solution of BA was prepared by dissolving BA (Merck, Germany) in a serum-free medium at concentrations ranging from 1 to 100 mM.

BC-SCs and MCF-7 cells were plated in 96-well plates at a density of 5 × 10^3^ cells per well. Following a 24-h incubation period, the medium was replaced. Subsequently, the cells were exposed to varying concentrations of BA ranging from 1 to 100 mM for 24 and 48 h. Untreated cells served as the control group. At the end of the periods, MTT (Invitrogen/GIBCO, Grand Island, NY, USA) solution was added to each well, and the cells were further incubated at 37 °C for 4 h in the dark. One hundred microliter of DMSO (Invitrogen/GIBCO, Grand Island, NY, USA) was added to each well and incubated for 15 min. The optical density was measured at 540 nm using a microplate reader (BIOTEK ELx808IU, Vermont, USA). All experiments were repeated three times.

### RNA Extraction, cDNA Synthesis, and Gene Expression Analysis by Real Time-qPCR

#### Total RNA isolation and cDNA Synthesis

Total RNA purification (EURx, Poland) was performed from the MCF-7 cells, breast cancer stem cells, and BA-treated breast cancer stem cells according to the manufacturer’s instructions. RNA concentrations were measured fluorometrically (Qubit Fluorometer, Thermofisher Scientific, USA). Then, the obtained RNA products were converted to complementary DNA (cDNA) using the cDNA synthesis kit (A.B.T.™, Turkey). Glyceraldehyde-3-phosphate dehydrogenase (GAPDH) was used as the normalization control.

#### miRNA Isolation and cDNA Synthesis

The miRNA purification (EURx, Poland) was performed from the MCF-7 cells, breast cancer stem cells, and BA-treated breast cancer stem cells according to the manufacturer’s instructions. RNA concentrations were measured fluorometrically (Qubit Fluorometer, Thermofisher Scientific, USA). Then, the obtained RNA products were converted to complementary DNA (cDNA) using the cDNA synthesis kit (A.B.T.™, Turkey). RNA U6 was used as the normalization control.

#### Quantitative Real-Time PCR

After completing the cDNA synthesis, the resulting cDNA samples from each group were used for the RT-qPCR reaction following the manufacturer’s protocol throughout the procedure (Blirt, Poland). Relative expression levels of human miR-127 and ZEB1, SNAIL, CDH1, ITGA5, ITGB1, COL1A1, VIM, and LAMA5 (Table 1) genes were estimated by Rotorgene Q5 plex + HRM Real-Time PCR device (Qiagen, Germany). After an initial denaturation at 95 °C for 3 min, the qRT-PCR reactions were carried out for 40 cycles, with each cycle consisting of 15 s at 95 °C, 30 s at 60 °C, and 30 s at 72 °C. Ct values (threshold cycle) were obtained post-qRT-PCR, using GAPDH and RNA U6 as the reference gene for control. The obtained results were analyzed using the 2^−ΔΔCt^ method. The primers (Suarge Biotechnology, Turkey) used in this study were presented in the table (Table 1).

**Table 1:** Primer sequences used for RT-qPCR analysis

Gene	Primer sequences (forward; reverse)
GAPDH	CACCCTGTTGCTGTAGCCATATTC; GACATCAAGAAGGTGGTGAAGCAG
ZEB1	CACCTCTCTGAGGCTGTG; CGTCATGTAAGCATCAATCAC
SNAIL1	ACAACCTGGAAGCTCAGT; TTAATACGCAGCAAACCAACA
ITGA5	TGATAGTAGTGTTAGTGATGCAAA; CAGCTAAAGGATTAATGGCAC
CDH1	ATCATTCACCCTTGGCAC; CATGGAAGCCATTGTCCT
ITGB1	CCTGATGCCTGTACACCTCTT; GCAGGCCGAGTACTGTTA
VIM	CTTGGGACAGAACCTAAAATG; GACGTCTCAGGTAGTGAAGAA
COL1A1	TGAGGGTACCTTTAGGCCAGA; CACTGCCAGAGAGACCATACC
U6	GCTTCGGCAGCACATATACTAAAAT CGCTTCACGAATTTGCGTGTCAT
hsa-miR-127-5p	GCGGTCGGATCCGTCTGA GAAAGAAGGCGAGGAGCAGATCGAGGAAGAAGACGGAAGAATGTGCGTCTCGCCTTCTTTCAGCCAAGC
LAMA5	AGAGTGAAGATGAGTTGACACCTT, CCAAGAGATCTCGATCACTGC

*GAPDH* glyceraldehyde 3-phosphate dehydrogenase; *ZEB1* zinc finger E-box binding homeobox 1; *SNAIL1* snail family transcriptional repressor 1; *ITGA5* integrin alpha-5; *CDH1* cadherin-1; *ITGB1* integrin subunit beta 1; *VIM* Vimentin; *COL1A1* Collagen, type 1, alpha 1; *LAMA5* laminin subunit alpha 5

### Statistical Analysis

Each experiment was repeated at least three times. The data obtained were evaluated as mean ± SD. The data was assessed using Student’s *t*-test and Tukey test. The significance threshold values of *p* < 0.05, *p* < 0.01, and *p* < 0.001 were used for differences. All statistical analyses were performed using GraphPad Prism 10.2.2 software.

## Results

### Boric Acid Reduced the CSC Population in MCF-7 Cells

**Fig. 1:**
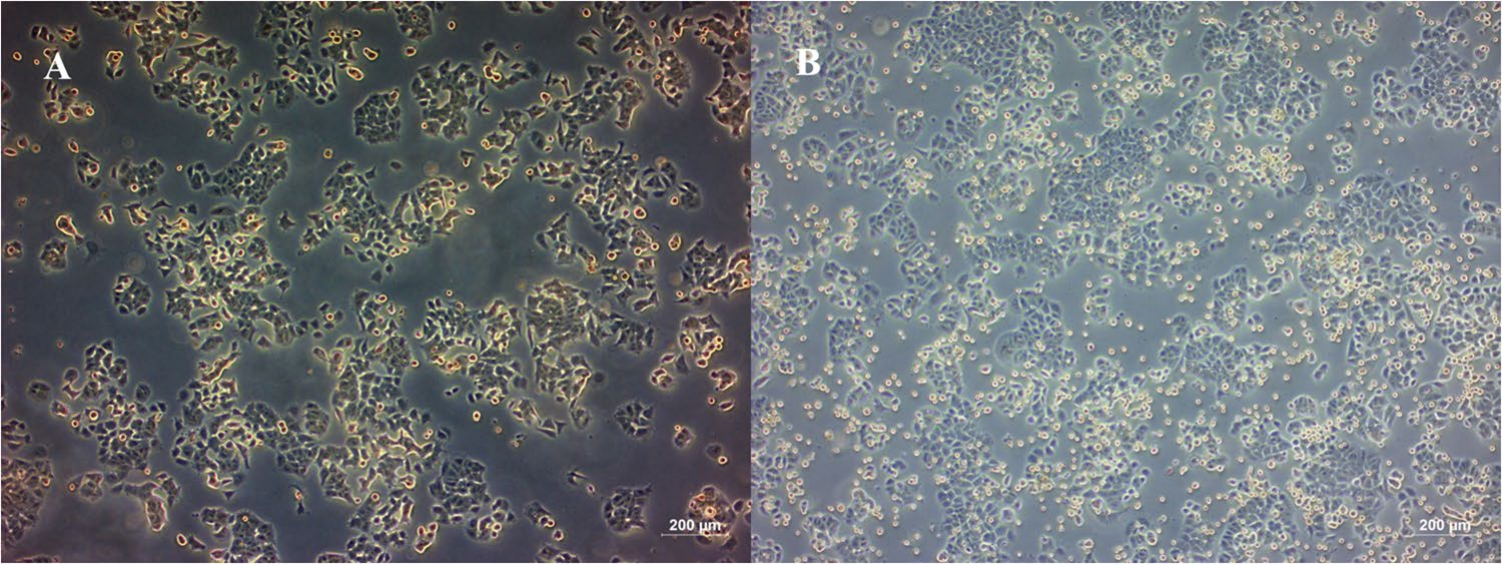
Morphology of MCF-7 cells and BC-SCs as viewed under an inverted microscope. **A** MCF-7 cells. **B** Formation of cell spheres by BC-SCs in serum-free medium (scale bar = 200 µm)

**Fig. 2:**
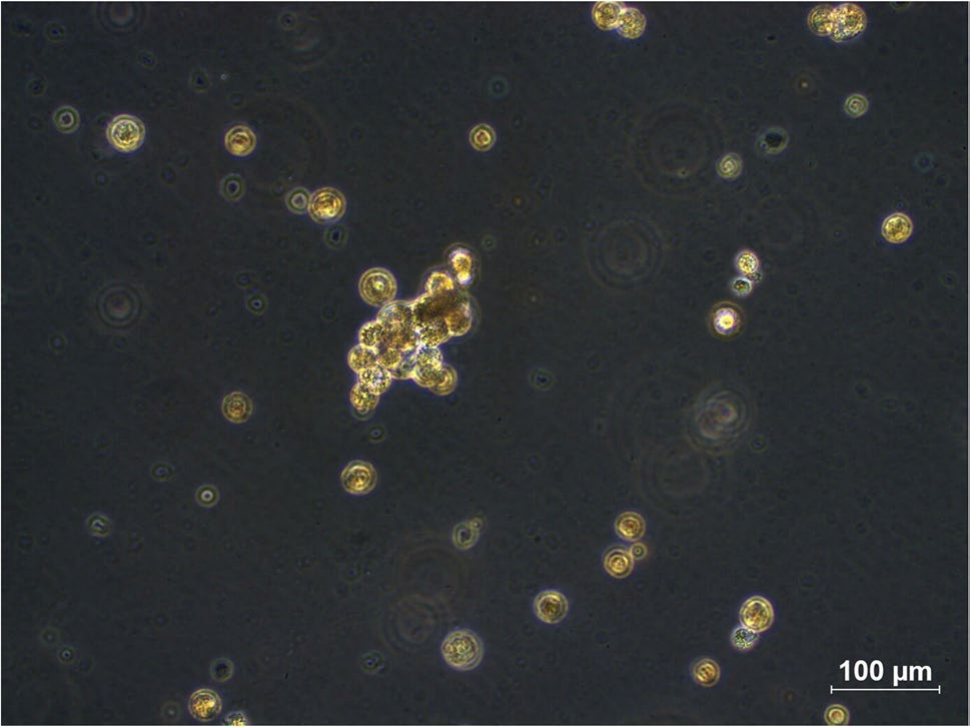
BC-SCs exhibited an enhanced capacity for sphere formation (scale bar = 100 µM)

**Fig. 3:**
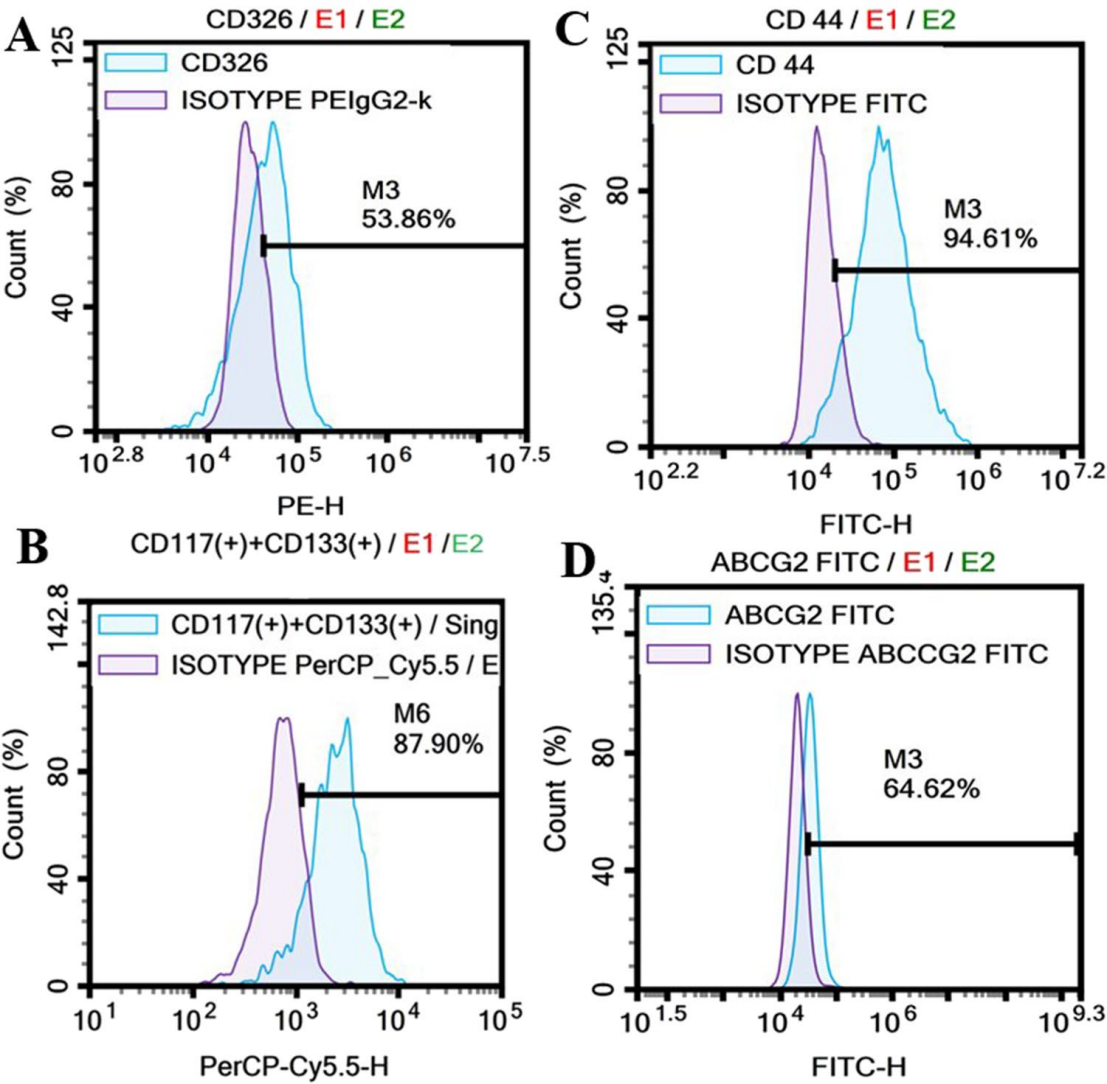
Flow cytometry analysis revealed the presence of BC-SCs. **A** CD326+ **B** CD117+/CD133+ **C** CD44+ **D** ABCG2 + cell populations on the surface of BC-SCs

Microscopic examinations showed that MCF-7 cells had fibroblast-like morphology (Fig. 1A). BC-SCs were also cultured (Fig. 1B) to confirm cancer stem cell properties. It was observed that cells could form spheroids of appropriate shape and size (Fig. 2). Flow cytometric analysis was performed to determine the purity of CD44 + , CD117 + , CD133 + , CD326 + , and ABCG2 + cells isolated by the MACS (Magnetic Activated Cell Sorting) method and to validate the method’s success. CD44 + , CD117 + , CD133 + , CD326 + , and ABCG2 + are shown in Fig. 3.

**Fig. 4:**
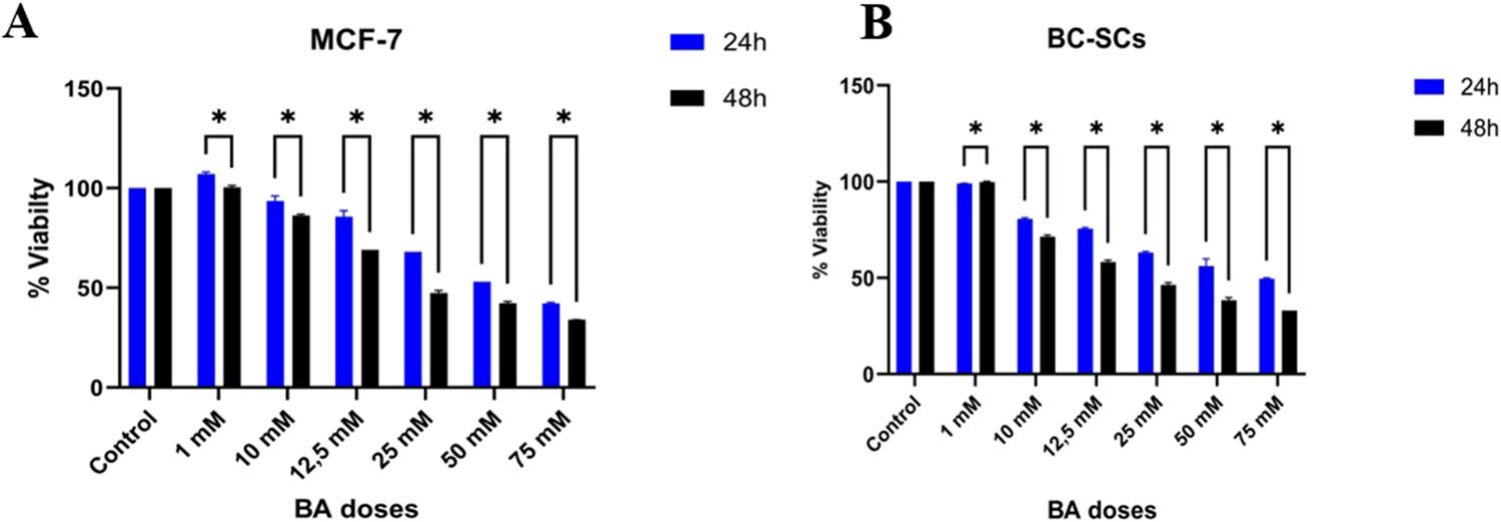
Impact of boric acid on cancer stem cell proliferation. Cells were cultured in a medium with varying concentrations of boric acid (0–100 mM), and cell viability was assessed using the MTT assay after 48 h. Data are presented as the mean ± standard deviation from three independent experiments (mean ± SD, *n* = 3). Statistical significance was denoted as *p < 0.05, **p < 0.01, and ***p < 0.001, while “ns” indicates no significant difference

It was observed that BA caused a decrease in cell viability of MCF-7 cells in a dose-dependent manner at 24 and 48 h. While no significant change in cell viability was observed at the 1 mM dose at 24 and 48 h, it was observed that other BA doses significantly reduced cell viability in both periods. The viability results of BA on MCF-7 cells at concentrations of 1–100 mM at 24 and 48 h are shown in the graph (Fig. 4). For BC-SCs, the IC50 dose was 41.27 mM for 48 h. Based on the gene expression study results, the time interval and BA dose that showed the most significant reduction in cell viability were selected. Consequently, 41.27 mM BA was used for the remainder of the study.

### BA Treatment Affected the Expression of miR127-5p and Metastasis Genes in BC-SCs

**Fig. 5:**
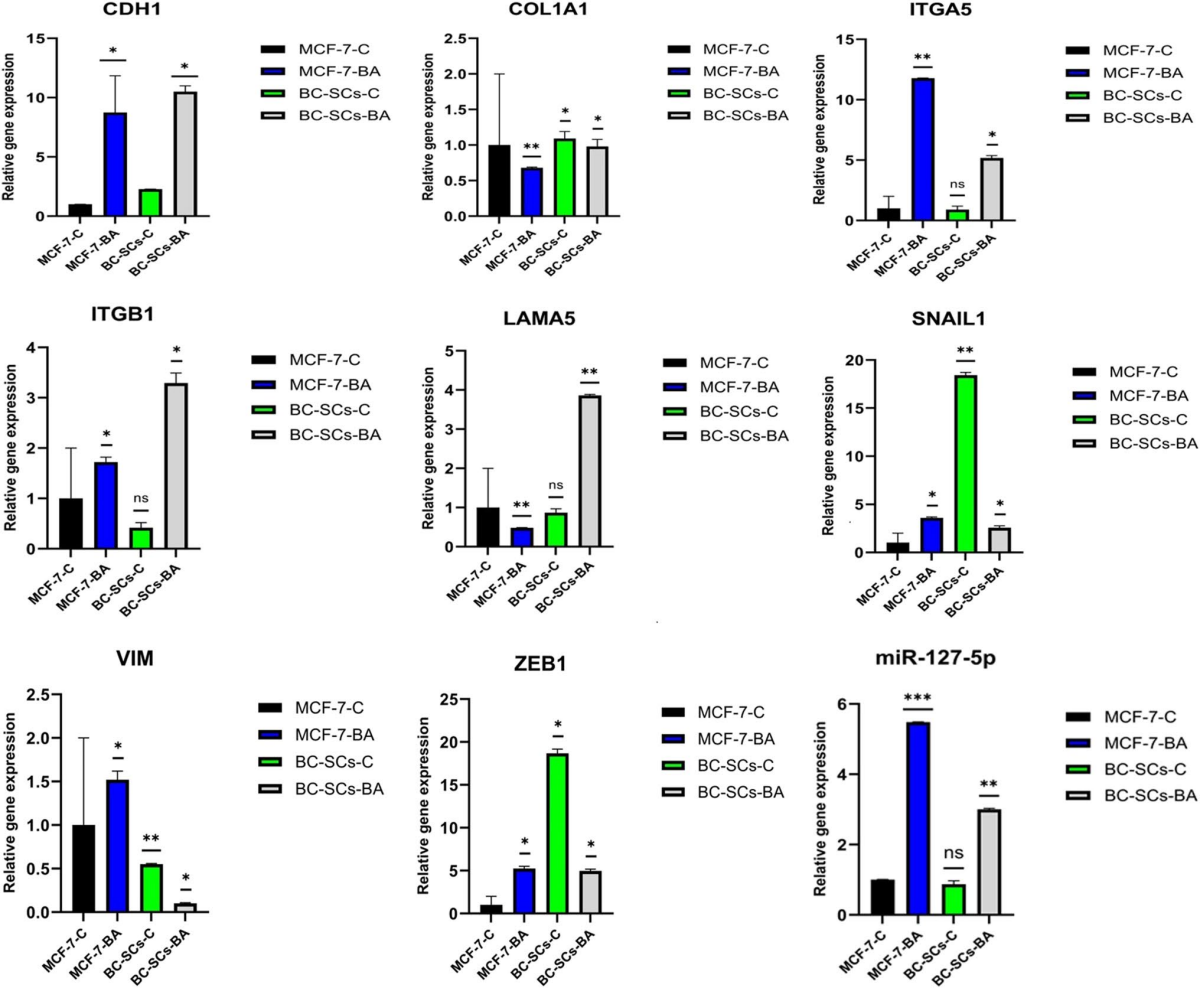
Expression profile of miR-127-5p and metastasis genes. In dose-treated BC-SCs, VIM and COL1A1 expression was significantly downregulated. Conversely, the expression levels of miR-127-5p, CDH1, ITGB1, ITGA5, ZEB1, SNAIL, and LAMA5 were notably up-regulated (mean ± SD, *n* = 3, *p < 0.05, **p < 0.01, and ***p < 0.001, with “ns” indicating no significant difference)

According to RT-qPCR results, miRNA127-5 expression was downregulated in both MCF-7 cells and BC-SCs. However, it was up-regulated in MCF-7-BA cells (*p* < 0.001) and BC-SC-BA (*p* < 0.01) cells after BA treatment. The expression of some metastasis genes, which are CDH1, ITGB1, ITGA5, ZEB1, SNAIL, and LAMA5 expressions of BC-SCs and BA-treated BC-SCs were significantly higher compared to MCF-7 cells; however, it was observed that COL1A1 and VIM expressions were decreased in BA-treated BC-SCs (Fig. 5).

## Discussion

Breast cancer stem cells (BC-SCs) play pivotal roles in breast tumor initiation, progression toward malignancy, and resistance to therapy [13]. They possess the unique capability of self-renewal and differentiation. Despite constituting a small fraction, approximately 2%, of the total breast tumor cell population, BC-SCs significantly contribute to metastatic spread, leading to elevated morbidity and mortality rates associated with breast cancer [14]. In our previous study and this study, we isolated and characterized breast cancer stem cells [12]. BC-SCs drive metastatic expansion, contributing significantly to the elevated morbidity and mortality associated with breast cancer. microRNAs (miRNAs) are pivotal in regulating CSC differentiation and self-renewal [15]. Growing evidence underscores the potential of miRNAs as promising therapeutic targets for breast cancer, exerting their effects by modulating BC-SC differentiation and self-renewal [16]. Downregulation of 127-5p may be an essential marker in breast cancer [17]. In our previous study, we examined the effect of boric acid (BA) on DNA double-break damage. In this study, we investigated the effect of BA on both metastasis and miR-127-5p expression, which is stated as a new marker. Since no previous study has investigated miR-127-5p expression in BC-SCs, our study is the first. Indeed, its expression increased approximately sixfold in breast cancer cells treated with BA, while its expression increased approximately fourfold in breast cancer stem cells. Boron is in the form of undissolved boric acid in the human body. It does not accumulate in tissues except bone tissue. While boric acid is rapidly absorbed, it is excreted from the body through urine with an average half-life of 1 day [18]. It has been reported that BA has an antiproliferative effect in various types of cancer [19–22]. Similar results have been shown in studies conducted with breast cancer [11, 23, 24]. As in our previous study, we obtained findings identical to those of the literature in this study [12]. The doses we used were the same as those in our previous studies. The doses used in studies on breast cancer and other types of cancer appear to be different from each other. This situation may be due to the biological and physiological differences of the cells used in the study. We used MCF-7 as the breast cancer cell line in this study and our previous study. In some studies, the effect of BA has been studied in different cells, such as MDA-MB-231 [9, 11, 23, 24]. Canturk et al. reported boric acid (at a concentration of 500 µM) cytotoxic effect on HL-60 (human promyelocytic leukemia cells) cells [25]. As a result, although the physiology of the cells is different, there may also be differences in which passage the cells are used and the techniques applied in the laboratory environment. Additionally, the biophysicochemical properties of the BA used may also differ.

We investigated whether the expression of genes responsible for metastasis in breast cancer stem cells isolated from MCF-7 cells changed with BA application. The effect of BA versus phenylboronic acid (PBA) on the migration of prostate and breast cancer cells showed that the impact of PBA rather than BA is more substantial than BA in targeting the metastasis of cancer cells [26]. In the study examining the effect of BA on the expression levels of lncRNA H19 and UCA1 and invasion genes in pancreatic cells, it was observed that the expressions of H19, UCA1, and CDH2 decreased while the expressions of CDH1 and TIMP1 increased.

Previous reports have reported that BA reduces the proliferation of breast cancer cells [9, 11, 27, 28]. Our previous study is the only study in the literature that examined the expression of metastasis genes in cancer stem cells after boric acid application [29]. E-cadherin (CDH1) is an essential regulator in the EMT process and can interact with multiple molecules and microRNAs through different mechanisms. miR-9 can initiate TGF-β-induced EMT and induce tumor metastasis in breast cancer by targeting the mRNA of E-cadherin [30]. In studies conducted with breast cancer cells, miRNAs responsible for the migration of cancer cells have been identified. In this study, CDH1 expression increased approximately 5-fold in cancer stem cells treated with BA compared to cancer stem cells without BA. Here, too, miR-127-5p may have helped increase CDH1 expression. Besides miR-127-5p, miRNAs responsible for cell proliferation, invasion, and migration may also be regulated.

In our previous study, while the expression of the transcription factor SNAIL increased in LC-SCs, no change was observed in the expression of ZEB1, another important transcription factor. While ZEB1 expression in breast cancer stem cells increased five-fold compared to MCF-7 cells without BA, it decreased almost 5-fold in cells applied to cancer stem cells without BA. While SNAIL expression increased 3-fold compared to BA-treated MCF-7 cells, it decreased 9-fold in BA-treated cells compared to non-BA-treated cancer stem cells. Cancer varies from tissue to tissue and can progress differently, even in a single tissue. Since breast and lung tissues have different genetic characteristics, the metastasis process also has different characteristics, and gene expression will naturally differ. CDH1 is regulated transcriptionally by ZEB1, ZEB2, Twist1, and SNAIL. miR-200 family members increase CDH1 transcription by directly targeting ZEB1 and ZEB2 mRNA [31]. miR-127-5p expression increased in BA-treated cancer stem cells. Yan et al. Their study on breast cancer miRNAs reported that one downregulated miRNA was miR-127-5p [32].

The expression of miR-127-5p was relatively low in both breast cancer and breast cancer stem cells without BA application. While miR-127-5p expression increased approximately 6-fold in breast cancer cells with BA application, it increased approximately threefold in breast cancer stem cells. DNA methylation and histone modification play critical roles in chromatin remodeling, development, and regulation of gene expression in diseases such as cancer [33]. miR-127-5p expression was up-regulated more than threefold by the chromatin-modifying drugs 5-aza-2′-deoxycytidine and 4-phenylbutyric acid. miR-127 is embedded in the CpG island and is expressed as part of the miRNA cluster in normal cells but is not expressed in cancer cells. miR-127-5p is involved in epigenetic silencing. BCL6, a potential target of miR-127, is translationally downregulated after treatment. DNA methylation and histone deacetylase inhibition can activate the expression of miRNAs that can act as tumor suppressors [34].

Integrins are in the steps of metastatic progression from cancer cell invasion, intravasation, extravasation, and distant colonization [35]. Our previous study showed that ITGA5 expression increased with BA treatment in lung cancer stem cells. Integrin α5 (ITGA5) expression increased in BA-treated breast and breast cancer stem cells. ITGA5 expression in invasive MDA-MB-468 cells was almost lost compared to other breast cancer cells. It was reported that ITGA5 was downregulated in breast cancer effusion compared to primary tumors; thus, it was stated that there may be a negative correlation between ITGA5 expression and breast cancer cell metastasis [36]. Integrins play an essential role in maintaining breast stem cells in normal breast tissue; deletion of integrins such as β1 impairs mammary gland development and function [37]. Knockdown of ITGB1 reduced the invasion and migration of Triple-negative breast cancer (TNBC) cells [38]. ITGB1 expression in breast cancer stem cells was significantly lower than in MCF-7 cells. While its expression increased approximately 1.7-fold in MCF-7 cells after BA application, it increased approximately 7-fold in BA-applied BC-SCs. COL1A1 is directly related to PTEN and ITGB1. Mechanistically, the increase in miR-127-5p expression after BA application may also indicate that BC-SCs slow the epithelial-mesenchymal transition process. However, since BA increases the expression of ITGB1, we cannot say the same. Increased ITGB1 expression may be indicative of induction of metastasis. miR-127-5p may perhaps induce ITGB1. Collagen is the main component of the ECM necessary for normal tissue function; it has crucial roles in maintaining stability and integrity in tissues and organs. Due to its importance in tissue development and maintenance of homeostasis, excessive collagen accumulation in the ECM can lead to various diseases. It has been stated that COL1A1 has a vital role in the metastasis of multiple types of cancer and the progression of other diseases [39]. COL1A1 expression was demonstrated by immunofluorescence staining in MCF7, T47D, MDA-MB-231, and BT54 cells [40]. Its expression decreased in both MCF-7 and BC-SCs treated with BA. COL1A1 is directly related to PTEN and ITGB1 [39]. Studies in breast cancer have reported that COL1A1 supports cell migration and invasion through various signaling pathways [40, 41]. COL1A1 supports ITGB1 and many other molecules in the invasion and migration of cancer cells. In ovarian cancer cells in which ITGB1 is knocked down, it reduced COL1A1-induced metastasis by partially suppressing the phosphorylation of AKT. ITGB1 is the molecule that mediates COL1A1 in the invasion and migration of cancer cells [42]. In this study, the partial decrease in the expression of COL1A1 and the partial increase in ITGB1 indicate that BA can induce these molecules in terms of metastasis.

Laminin is the main component of the basement membrane. Laminins are essential in tumor cell proliferation, angiogenesis, migration, and metastasis. LAMA5 is vital in the migration of many cancers, including breast carcinoma, fibrosarcoma, and colon cancer. LAMA5 is a molecular target of KRAS-mutated colorectal cancer cells and is overexpressed in the tumor microenvironment (TME) [43]. In this study, LAMA5 expression decreased in MCF-7 cells after BA application and increased in BC-SCs. Expression of LAMA5 in hepatic metastases has been associated with increased vessel density, which links the expression of laminin chains to angiogenesis [44]. It is related to the expression of CD44 and LAMA5, which are markers associated with stem cell maintenance [45]. LAMA5 is also directly related to the COL1A1 molecule [46]. We cannot say that miR-127-5p, which is up-regulated in BC-SCs with the effect of BA, affects LAMA5 expression. LAMA5 remains active due to the influence of Wnt-β Katenin, Hedgehog, and other signaling pathway molecules and miRNAs. BA does not seem to affect the expression of this molecule.

Vimentin (VIM) is an epithelial-mesenchymal transition marker expressed in some types of cancer, including breast cancer [47]. While VIM expression was up-regulated in BA-treated MCF-7 cells, it was downregulated in BA-treated BC-SCs. VIM is associated with tumor growth, invasion, and poor prognosis. It is also considered a regulator of focal adhesions (FAs) and motility. VIM is activated by assembling into focal adhesions. Adding VIM to focal adhesions is crucial to cell adhesion strength [46].

With this study, we demonstrated the effect of BA on the expression of miR-127-5p and genes responsible for metastasis in both MCF-7 cells and BC-SCs. BA increased the expression of miR-127-5p in breast cancer stem cells. However, it cannot be said that it has the same effect on metastasis genes. Studies of BA and BA-derived compounds on cancer stem cells are limited. Our previous studies showed the impact of BA on the DNA double-break repair mechanisms and metastasis of lung and breast cancer stem cells. BA alone does not appear to be effective in cancer stem cells. The limitations of this study include not being able to perform combination therapy with another chemotherapeutic agent, not being able to study different miRNAs and signaling pathways that affect cancer stem cells, and not being able to study these factors in 3D culture and in vivo. Since the tumor microenvironment is a separate and complex architectural structure, we have a long way to go. Since we consider cancer stem cells to be the critical building blocks of this architectural structure, we hope our studies’ results will guide researchers working in this field.

## Data Availability

All data is contained within the article.
